# Oral Chemical Burn Due to Occupational Exposure to Potassium Hydroxide: An Unusual Case Report

**DOI:** 10.1111/scd.70289

**Published:** 2026-11-06

**Authors:** Camila Barcellos Calderipe, Carla Isabelly Rodrigues‐Fernandes, Thaís Cristina Esteves‐Pereira, Ivan José Correia Neto, Rogério De Andrade Elias, Pablo Agustin Vargas, Marcio Ajudarte Lopes, Alan Roger Santos‐Silva

**Affiliations:** ^1^ Department of Oral Diagnosis Dental School University of Campinas (UNICAMP), Piracicaba Piracicaba São Paulo Brazil

**Keywords:** alkali, occupational exposure, photobiomodulation, potassium hydroxide

## Abstract

**Objective:**

To report an uncommon case of oral chemical burn caused by occupational exposure to potassium hydroxide (KOH) managed with photobiomodulation (PBM).

**Case Report:**

A 50‐year‐old man experienced occupational exposure to KOH while working in a food factory. The accident occurred during maintenance of a pipeline that should have been empty; however, it was still carrying a 50% KOH solution. The patient presented with severe pain and impaired speech, nutrition, and hydration due to the extent of the lesions. Clinical examination revealed diffuse ulcerations affecting the lower labial semimucosa, upper and lower labial mucosa, and dorsal tongue. A clinical diagnosis of KOH‐induced chemical burn was established, and daily PBM therapy was initiated using red (660 nm) and infrared (850 nm) light‐emitting diodes (LEDs) to provide pain control and promote mucosal repair. Rapid improvement of signs and symptoms was observed throughout treatment, with complete mucosal healing achieved after eight sessions of PBM.

**Conclusion:**

This report describes, to the best of our knowledge, the first documented case of oral burn caused by KOH in an occupational setting, highlighting the severity of alkali‐induced lesions and the therapeutic potential of PBM therapy.

## 1 Introduction

According to the International Labour Organization (ILO), a work‐related accident is an unexpected event arising from or related to work that results in bodily injury, illness, or death [1]. Occupational accidents involving chemical agents can cause mucocutaneous burns, resulting in tissue and functional impairment [2, 3, 4]. Alkaline compounds have the potential to cause severe burns due to their capacity for progressive tissue damage after exposure, although such agents are not the most frequently associated with these accidents [2, 3, 5]. In this context, potassium hydroxide (KOH), in highly concentrated solutions, has been used in industrial settings for cleaning processes due to its corrosive properties [6].

The literature on KOH‐related oral burns is scarce and largely limited to domestic settings, with insufficient clinical documentation and limited detail regarding therapeutic management [7, 8]. To our knowledge, no previous reports have described oral burns associated with occupational exposure to KOH. This occurrence is relevant not only due to its scarcity but also to the potential functional consequences, such as impairment of speech and nutrition associated with severe pain. Therefore, the present study aims to report an uncommon case of oral chemical burn caused by occupational exposure to KOH managed with photobiomodulation (PBM).

## 2 Case Report

This case report was approved by the local Research Ethics Committee (approval number: CAAE: 93698225.2.0000.5418) and was prepared in accordance with the CARE (CAse REports) guidelines [9].

A 50‐year‐old man presented to the Oral Medicine Service one week after occupational exposure to potassium hydroxide (KOH). The patient worked in a food processing plant, and the accident occurred during maintenance of a pipeline used for the flow of a 50% KOH solution. The substance was used to clean industrial preparation equipment, and the pipeline should have been empty at the time of the procedure.

The patient was wearing personal protective equipment (PPE) when KOH splashed onto his gloves during maintenance. At that moment, he removed his protective face mask, and the substance splashed into his oral cavity. Subsequently, the patient used a topical burn spray available in his work sector. According to the history provided, application of the spray pushed the substance further into the oral cavity, after which the patient expelled the contents. The patient subsequently sought care at the medical unit of the company, where he was informed that the use of the spray had already neutralized the substance. He was not physically examined and was discharged home.

In the seven days following the accident, he sought two additional medical consultations due to pain. On these occasions, he received an intramuscular injection of a long‐acting nonsteroidal anti‐inflammatory drug (ketoprofen and diclofenac), along with a sublingual prescription for analgesia (ketorolac 20 mg every 6 h).

On admission to our Oral Medicine Service, the patient appeared markedly prostrate, and his history was reported by a companion. The patient was unable to communicate clearly and required a straw for both nutrition and hydration due to the extent of the lesions. Severe pain persisted despite analgesic use, with a score of 7 on the Visual Analog Scale (VAS), and he remained absent from work. His past medical history was unremarkable.

Clinical examination revealed very painful ulcerations involving the lower labial semimucosa, upper and lower labial mucosa, and dorsal tongue. During clinical management, pseudomembranes, bleeding points predominantly on the lips, and crust formation on the lower labial semimucosa were observed. Although the ulcerations were extensive, the lesion margins were well‐defined. A diagnosis of KOH‐induced oral chemical burn was established. Daily PBM therapy (excluding weekends) was proposed to provide analgesia and accelerate healing. The treatment was performed using the THOR LX2 device with the LED Lollipop probe (Figure 1), composed of 18 red LEDs (660 nm, 80 mW × 18 LEDs) and 23 infrared LEDs (850 nm, 60 mW × 23 LEDs), totaling 41 emitters with 2800 mW combined output power. Each session involved 180‐second irradiation to the lower labial semimucosa and mucosa, upper labial mucosa, and dorsal tongue. The probe was positioned in gentle contact, perpendicular to the tissue, delivering simultaneous, continuous emission of red and infrared light, with 504 J of energy delivered per point. The beam area parameter was not available from the manufacturer for the LED Lollipop probe, which prevented the calculation of irradiance, fluence, and photon fluence. Although an estimate of the application area over the lesions could be performed, the lack of information on the spatial distribution of the LEDs within the probe, which is three‐dimensional, would prevent obtaining reliable values. These reported dosimetric parameters are in accordance with the recommendations of the Jenkins & Carroll (2011) guidelines [10].

**FIGURE 1:**
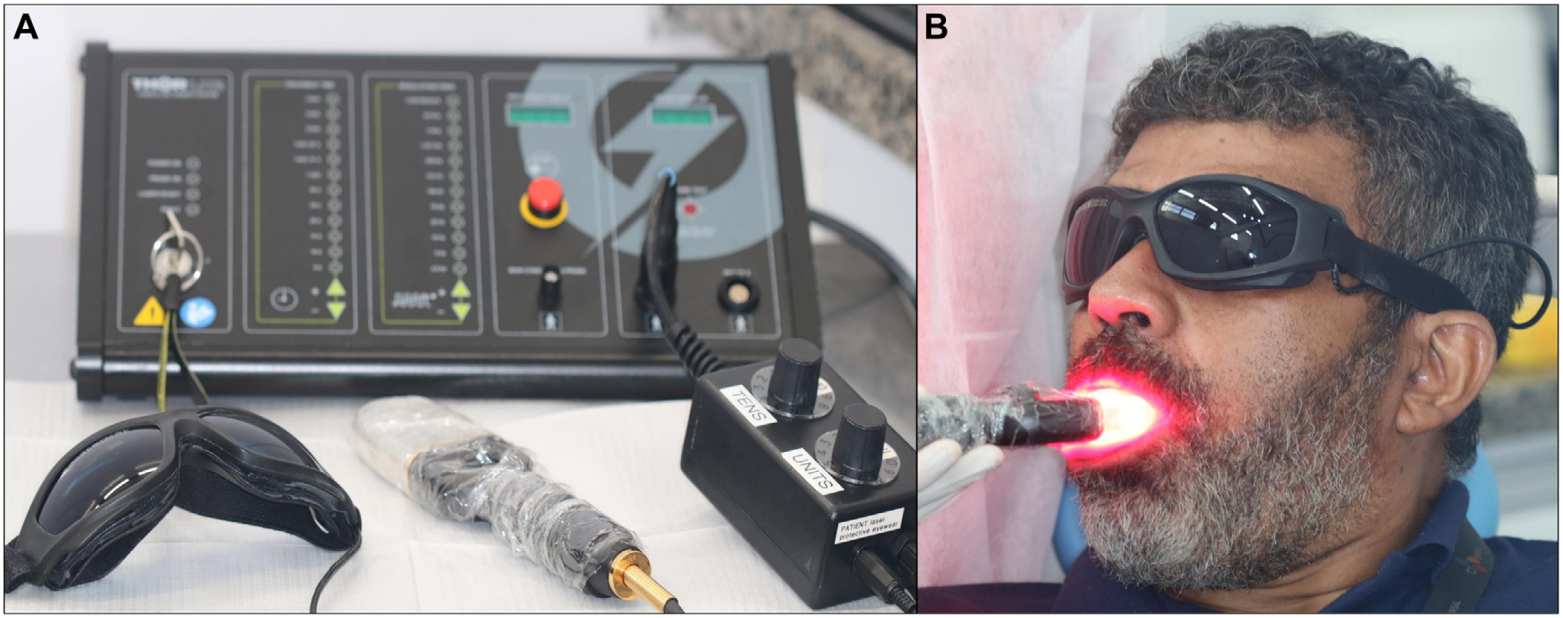
A: THOR LX2 device with LED Lollipop probe; B: Application of PBM therapy on the patient's dorsal tongue.

Following a single session, the patient returned without prostration, with reduced pain (VAS score 5), and was able to drink water without a straw. After three PBM sessions, the patient reported pain control, normal oral intake, and complete recovery of speech. Following four sessions, he discontinued the analgesic initially prescribed and returned to work. Throughout the treatment, rapid improvement was observed, with complete mucosal healing achieved after eight sessions of PBM therapy over 13 days. The patient was last reassessed one year after the accident, with no evidence of late sequelae, and was subsequently discharged from the service. (Figure 2; Table 1).

**FIGURE 2:**
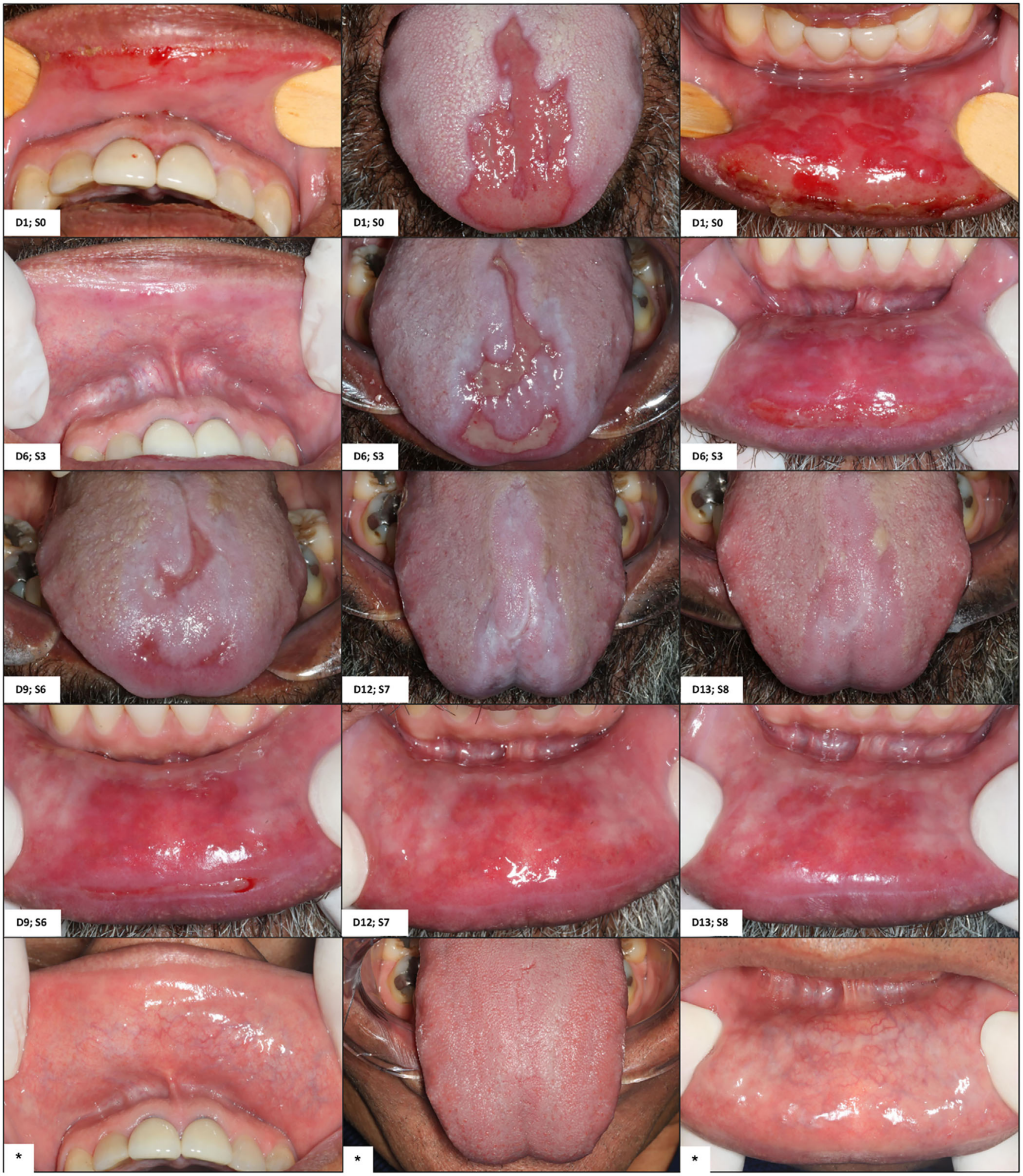
Clinical course of KOH‐induced chemical burns in the patient. D: days since admission to the Oral Medicine Service; S: number of PBM sessions; *: one year after the accident.

**TABLE 1:** Timeline of clinical course.

DAY	D. OMS	VAS	EVENT
1		8	Occupational exposure to KOH
1–7		7	Three medical consultations; intramuscular injections of long‐acting nonsteroidal anti‐inflammatory drugs (ketoprofen and diclofenac); prescription for sublingual analgesia (ketorolac 20 mg every 6 h).
8	1	7	First consultation at the Oral Medicine Service; diagnosis established; no signs of epithelialization; first PBM session.
9	2	5	After one PBM session, no prostration, reduced pain, and ability to drink water without a straw; second PBM session.
10, 11	3, 4	4	Weekend
12	5	3	After two PBM sessions, tolerating oral intake without major difficulties; third PBM session.
13	6	0	After three PBM sessions, pain control achieved, normal oral intake, and complete recovery of speech; complete healing of the upper lip; fourth PBM session.
14	7	0	After four PBM sessions, ketorolac discontinued and work activities resumed; fifth PBM session.
15, 16	8, 9	0	Progressive reduction of lesions on the tongue and lower labial mucosa; sixth and seventh PBM sessions.
17, 18	10, 11	0	Weekend
19	12	0	After seven PBM sessions, complete healing of the lower labial mucosa, although erythema persisted; small residual lesion on the tongue; eighth PBM session.
20	13	0	After eight PBM sessions, complete healing of the tongue and, consequently, of the oral mucosa.

Abbreviations: D.OMS, days since admission to the oral medicine service; VAS, visual analog scale.

## 3 Discussion

In 2023, the ILO estimated that 395 million workers worldwide experienced nonfatal occupational accidents, and 2.93 million workers died due to work‐related factors [11]. The prevention and initial management of occupational accidents involving caustic agents require a structured approach encompassing the proper use of PPE, worker training, effective emergency response protocols, and the availability of trained first‐aid personnel. In the present case, despite the use of PPE, exposure occurred due to failures in handling procedures, associated with inadequate management of immediate post‐exposure measures, highlighting the importance of strict adherence to safety protocols.

According to Wang et al. (2018), in a local epidemiological investigation, chemical burns predominantly occurred in men engaged in occupational activities involving improper handling of chemical agents [3]. From an occupational perspective, and in other possible contexts of exposure to chemical substances, acidic compounds have most frequently been associated with chemical burns [2, 3]. However, alkalis such as KOH can cause more severe injuries than those induced by acids [12]. While acids promote coagulative necrosis, alkalis induce liquefactive necrosis, allowing deeper tissue penetration and severe internal damage [4].

Alkali burns may continue to penetrate and damage tissues for hours or even days after the initial exposure if not promptly managed [5]. Alkaline substances cause burns by saponifying cell membrane lipids and denaturing proteins, allowing rapid tissue infiltration. Unlike acid burns, which often form a protective coagulum barrier, alkali burns do not, resulting in progressive tissue destruction long after the initial contact [4, 12].

Our literature search found few reports of accidental exposure to KOH involving the oral cavity. El‐Hawary [7] recently described a case of accidental household exposure to KOH that resulted in a tongue burn. The patient, a 7‐year‐old child, chewed solid KOH used as a laundry agent after dilution. The burn partially impaired tongue mobility, and due to inadequate initial management, progressed to complete ankyloglossia. It is noteworthy that the patient was referred to El‐Hawary [7] one year after the incident for surgical repair of the ankylosis. Similarly, Massa and Ludemann [8] reported a domestic accident involving a 2‐year‐old child who ingested a KOH‐containing product used for pH regulation in soil cultivation. The accident caused burns in the oral cavity, pharynx, and esophagus. Management included analgesics, antireflux therapy, antibiotics, and corticosteroids [8]. As observed in El‐Hawary's [7] report, no clinical images of the lesions were provided.

Previously reported cases of oral burns caused by sodium hydroxide (NaOH), another alkaline compound, have included photographic documentation of both initial lesions and clinical course during treatment. Such documentation enhances understanding of lesion extent, clinical impact, and healing progression over time [13, 14]. In this context, the present report is, to the best of our knowledge, the first to describe oral burns associated with occupational exposure to KOH and likely the first to provide photographic documentation of oral KOH burns in any setting.

Strongly caustic substances such as KOH require rigorous protective measures, including gloves, impermeable clothing, eye or face protection, and, when indicated, respiratory protection [15, 16]. Adherence to the proper use of PPE is linked to workers' knowledge and supervision, emphasizing the importance of training and monitoring programs [15]. Inadequate risk perception has been linked to the occurrence of occupational accidents and injuries [17, 18], underscoring the need for educational strategies that promote awareness of workplace hazards. The effectiveness of PPE ultimately depends on both use and appropriate actions following accidental exposure.

Safety Data Sheets (SDSs) are mandatory for all chemical products in the workplace. They are essential for promptly identifying the responsible agent and initiating immediate intervention to minimize tissue damage. The implementation of rapid‐response protocols, including the availability of washing stations and emergency showers, is crucial to reducing injury severity in exposures to water‐manageable substances such as KOH [4, 19]. Initial management consists of the immediate removal of the chemical agent and copious irrigation with running water for at least 20–30 min, along with the removal of contaminated clothing [19]. After decontamination, the extent and depth of the lesion should be assessed, and systemic complications monitored [4, 19]. In cases of ingestion, inhalation, or ocular contact, additional specialized evaluation is required, and in extensive exposures, systemic toxicity due to absorption should be considered [4]. Pain management should be optimized, and severe cases may require referral to specialized units for possible debridement or skin grafting [4].

Continuous wound hygiene is essential to prevent secondary infection, to protect the area with dressings when applicable, and to document the healing process [4, 19]. In this context of local wound care, PBM therapy has emerged as a promising therapeutic modality for managing mucosal and skin lesions. Studies have demonstrated that this therapy can significantly reduce pain levels and accelerate healing by activating growth factors and modulating the inflammatory response [20, 21, 22, 23], making it a relevant therapeutic option to optimize recovery and patient comfort.

Given that PBM was initiated only on the eighth day after the accident, it is not possible to determine its precise contribution to accelerating the healing process. Moreover, the limited availability of data in the literature regarding the spontaneous course of alkaline chemical burns in the oral mucosa limits direct comparisons. Nainggolan et al. [14] reported a case of an extensive oral burn caused by NaOH, in which healing occurred within 14 days in the upper labial mucosa, 42 days in the lower labial mucosa, and 56 days in the tongue; however, it should be considered that the repair time is directly related to the extent of the lesions. Nevertheless, in the present case, the early analgesic response observed after the first PBM session suggests a potential therapeutic benefit of this modality, particularly in improving patient comfort during the healing process.

Despite the potential severity of oral chemical burns caused by alkaline agents, the oral mucosa exhibits a superior reparative capacity compared to the skin, characterized by a reduced inflammatory response and limited scar formation, which may contribute to favorable outcomes [24]. Nevertheless, these lesions may progress to late complications, including fibrosis, oral contracture, dysphagia, and dysarthria, depending on the extent of the lesion and the management provided [7, 24].

The present report describes, to the best of our knowledge, the first documented case of oral burn caused by KOH in an occupational setting. This case highlights the severity of alkali‐induced lesions, which can result in communication and feeding impairment and severe pain. PBM therapy proved effective, particularly in promoting analgesia, leading to complete functional recovery within a short period. This case reinforces the importance of strict preventive measures in occupational environments involving caustic substances, including proper PPE use, continuous worker training, and the availability of rapid‐response protocols to minimize damage in cases of accidental exposure.

## Author Contribution

**Camila Barcellos Calderipe**: contributed to the clinical care and management of the patient; the interpretation of the clinical course; the conception and planning of the study; drafting the manuscript and critically revising it for important intellectual content; approved the final version to be published; and agreed to be accountable for all aspects of the work. **Carla Isabelly Rodrigues‐Fernandes**: contributed to the clinical care and management of the patient; the interpretation of the clinical course; drafting the manuscript and critically revising it for important intellectual content; approved the final version to be published; and agreed to be accountable for all aspects of the work. **Thaís Cristina Esteves‐Pereira**: contributed to the clinical care and management of the patient; the interpretation of the clinical course; drafting the manuscript and critically revising it for important intellectual content; approved the final version to be published; and agreed to be accountable for all aspects of the work. **Ivan José Correia Neto**: contributed to the clinical care and management of the patient; the interpretation of the clinical course; drafting the manuscript and critically revising it for important intellectual content; approved the final version to be published; and agreed to be accountable for all aspects of the work. **Rogério De Andrade Elias**: Contributed to the clinical care and management of the patient; the interpretation of the clinical course; drafting the manuscript and critically revising it for important intellectual content; approved the final version to be published; and agreed to be accountable for all aspects of the work. **Pablo Agustin Vargas**: Contributed to the interpretation of the clinical course; drafting the manuscript and critically revising it for important intellectual content; approved the final version to be published; and agreed to be accountable for all aspects of the work. **Marcio Ajudarte Lopes**: Contributed to the interpretation of the clinical course; drafting the manuscript and critically revising it for important intellectual content; approved the final version to be published; and agreed to be accountable for all aspects of the work. **Alan Roger Santos‐Silva**: Contributed to the clinical care and management of the patient; the interpretation of the clinical course; the conception and planning of the study; drafting the manuscript and critically revising it for important intellectual content; approved the final version to be published; and agreed to be accountable for all aspects of the work.

## Funding

The authors have nothing to report.

## Ethical Statement

The study was approved by the local Research Ethics Committee, in accordance with institutional and international ethical standards.

## Consent

Written informed consent was obtained from the patient, authorizing the use and publication of clinical data and photographic records related to the case.

## Conflicts of Interests

The authors declare no conflicts of interest.
